# A New Epoxy-cadinane Sesquiterpene from the Marine Brown Alga *Dictyopteris divaricata*

**DOI:** 10.3390/md7040600

**Published:** 2009-11-17

**Authors:** Yuan-Yuan Qiao, Nai-Yun Ji, Wei Wen, Xiu-Li Yin, Qin-Zhao Xue

**Affiliations:** Yantai Institute of Coastal Zone Research, Chinese Academy of Sciences, Yantai 264003, China; E-Mails: qq697@126.com (Y.-Y.Q.); wenwei_500@sina.com (W.W.); xlyin@yic.ac.cn (X.-L.Y.); qzxue@yic.ac.cn (Q.-Z.X.)

**Keywords:** Dictyopteris divaricata, sesquiterpene, cadinane

## Abstract

A new epoxy-cadinane sesquiterpene, 4*β*,5*β*-epoxycadinan-1*β*-ol (**1**), and six known cadinane sesquiterpenes: cadinan-1,4,5-triol (**2**), 4*α*,5*β*-dihydroxycubenol (**3**), cubenol (**4**), cadinan-3-ene-1,5-diol (**5**), cubenol-3-one (**6**), and torreyol (**7**), were isolated from a sample of marine brown alga *Dictyopteris divaricata* collected off the coast of Yantai (China). Their structures were established by detailed MS and NMR spectroscopic analysis, as well as comparison with literature data.

## 1. Introduction

Marine brown algae of the genus *Dictyopteris* (Dictyotales, Dictyotaceae) have been found to be a rich source of structurally unique sesquiterpenes, including cadinane, selinane, germacrane, and other rearranged skeleton types [1–9]. Continuing chemical investigation of the secondary metabolites of *D. divaricata* collected off the coast of Yantai has led to the isolation of one new cadinane sesquiterpene, 4*β*,5*β*-epoxycadinan-1*β*-ol (**1**), and six known cadinane sesquiterpenes: cadinan-1,4,5-triol (**2**) [5], 4*α*,5*β*-dihydroxycubenol (**3**) [6], cubenol (**4**) [6], cadinan-3-ene-1,5-diol (**5**) [5], cubenol-3-one (**6**) [6], and torreyol (**7**) [6]. The isolation of compounds **1**–**7** and structural determination of compound **1** are presented.

## 2. Results and Discussion

**Figure 1.**
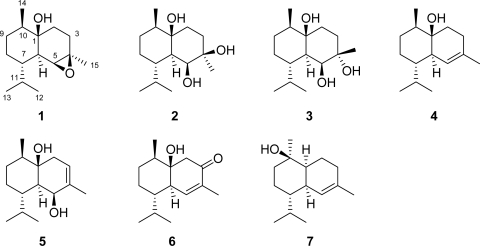
Structures of compounds **1**–**7**.

The dried and powdered alga *D. divaricata* was extracted with a mixture of CHCl_3_ and MeOH (1:1, v/v). The concentrated extracts were partitioned between H_2_O and EtOAc. The EtOAc-soluble fraction was purified by a combination of silica gel, reversed-phase silica gel, and Sephadex LH-20 column chromatography, as well as preparative TLC, to yield compounds **1**–**7** (Figure 1).

**Figure 2.**
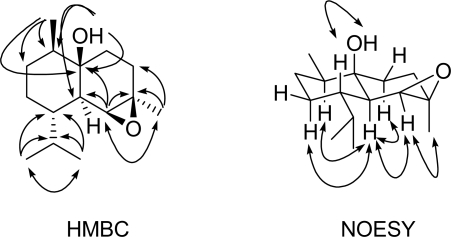
HMBC and NOESY correlations of compound **1**.

Compound **1** was obtained as a colorless oil. The IR absorption at *v*_max_ 3471 cm^−1^ indicated the presence of a hydroxyl group in the molecule. The molecular formula was determined as C_15_H_26_O_2_ on the basis of HRESIMS (*m*/*z* 261.1825 [M+Na]^+^, calcd. for C_15_H_26_O_2_Na, 261.1830), suggesting three degrees of unsaturation. The ^1^H-NMR spectrum displayed one methyl singlet, three methyl doublets, and two broad singlets attributed respectively to an oxygenated methine and a hydroxyl group. The ^13^C-NMR spectrum along with the DEPT and HSQC experiments revealed the presence of four methyls, four methylenes, five methines, and two quaternary carbon atoms. A detailed comparison of the above spectral data with those reported for cadinan-1,4,5-triol (**2**) revealed that **1** differed from **2** mainly at C-4 and C-5 [5], so **1** could be a dehydrated product of **2** between the hydroxyl groups at C-4 and C-5 according to the upfield-shifted C-4, C-5, and H-5 [10]. The ^1^H-^1^H COSY correlations as shown in Table 1 and the observed HMBC (Figure 2) correlations from H-12 to C-7, C-11, and C-13, from H-13 to C-7, C-11, and C-12, from H-14 to C-1, C-9, and C-10, from H-15 to C-3, C-4, and C-5, from H-5 to C-1, C-4, C-6, and C-15, and from HO-1 to C-1, C-6, and C-10 confirmed the gross structure of **1**.

The relative stereochemistry of **1** was determined by analysis of NOESY correlations (Figure 2) and coupling constants. The NOESY correlations between H-6 and H-2a, H-8a, H-10 indicated them to be axial and on the same face of the molecule. H-5 was located on the same face of H-6 and C-15 based on the NOESY correlation between H-5 and H-6, H-15 and the little coupling constant (only broad singlet) of H-5, while the large coupling constant (11.6 Hz) of H-6 suggested H-6 and H-7 to be opposite. The observed NOESY correlation between HO-1 and H-7 indicated them to be located on the same face of the molecule. The above evidence established the structure of **1** to be 4*β*,5*β*-epoxycadinan-1*β*-ol.

The structures of known compounds **2**–**7** were confirmed by detailed NMR data comparison with those in literature [5,6]. Compound **6** is firstly reported from *D. divaricata*, while **2**–**5**, **7** have been isolated from this species before [5,9,11]. When we tried to purify compound **1** by preparative TLC, **2** was by-produced. So, compound **2** may be an artifact, though it has been isolated from a different fraction (fraction VIII). Compound **1** represents a new addition to the molecular diversity of cadinane sesquiterpenes, which may be a key intermediate in the biosynthesis from **4** to **2** and **3**.

## 3. Experimental Section

### 3.1. General

NMR spectra were recorded in CDCl_3_ at 500 and 125 MHz for ^1^H and ^13^C, respectively, on a Bruker Avance 500 MHz NMR spectrometer with TMS as internal standard. Mass spectra were determined on a VG Autospec 3000 mass spectrometer. IR spectrum was obtained on a JASCO FT/IR-4100 Fourier Transform InfraRed spectrometer. Optical rotation was measured on a JASCO P-1020 polarimeter. Column chromatography was performed with silica gel (200–300 mesh, Qingdao Haiyang Chemical Co., Qingdao, China), RP-18 reversed-phase silica gel (YMC), and Sephadex LH-20 (Pharmacia). TLC was carried out with precoated silica gel plates (GF-254, Qingdao Haiyang Chemical Co., Qingdao, China). All solvents were of analytical grade.

### 3.2. Algal Material

The brown alga *Dictyopteris divaricata* was collected off the coast of Yantai (lat. 37°31′15″N, long. 121°26′59″E), Shandong Province, China, in July 2008. It was identified by one of the authors (Nai-Yun Ji) and a voucher specimen (MBA0807) has been deposited at the Bio-Resource Laboratory of Yantai Institute of Coastal Zone Research, Chinese Academy of Sciences.

### 3.3. Extraction and Isolation

Dried and powdered alga *D. divaricata* (2 kg) was extracted with a mixture of CHCl_3_ and MeOH (2 L, 1:1, v/v). The concentrated extract was partitioned between H_2_O and EtOAc. The EtOAc-soluble fraction (90 g) was fractioned by silica gel column chromatography (petroleum ether (PE)/EtOAc gradient) to give ten fractions, I-X. Fraction III, eluted with PE/EtOAc (50:1), was further purified by silica gel column chromatography (PE/EtOAc 10:1) to afford **4** (*ca.* 30 g). Fraction IV, eluted with PE/EtOAc (20:1), was further purified by silica gel and Sephadex LH-20 (CHCl_3_/CH_3_OH) column chromatography to afford *4β,5β-epoxycadinan-1β-ol* (**1**, 0.8 mg). Colorless oil; [*α*]^21^_D_–8.2° (c=0.13, CHCl_3_); IR (KBr) cm^−1^: 3471, 2954, 2923, 2877, 1450, 1392, 976; ^1^H-NMR (CDCl_3_, 500 MHz) and ^13^C-NMR (CDCl_3_, 125 MHz): see Table 1; HRESIMS *m*/*z*: 261.1825 [M+Na]^+^, calcd. for C_15_H_26_O_2_Na, 261.1830. Fraction VII, eluted with PE/EtOAc (2:1), was further purified by Sephadex LH-20 (CHCl_3_/CH_3_OH) and RP-18 (CH_3_OH/H_2_O 3:1) column chromatography and preparative TLC (PE/EtOAc 3:1) to afford **3** (11.0 mg), **5** (2.1 mg), **6** (14.0 mg), and **7** (8.0 mg). Fraction VIII, eluted also with PE/EtOAc (2:1), was further purified by Sephadex LH-20 (CHCl_3_/CH_3_OH) and RP-18 (CH_3_OH/H_2_O 3:1) column chromatography and preparative TLC (PE/EtOAc 1:4) to afford **2** (20.7 mg).

## Figures and Tables

**Table 1.** ^1^H and ^13^C-NMR data and ^1^H-^1^H COSY correlations of compound **1** (in CDCl_3_, *δ* values, *J* in Hz).

***No.***	***δ*_C_**	***δ*_H_**	**^1^H-^1^H COSY**
1	71.4 s		
2a	31.7 t	1.16 (ddd, 13.4, 13.1, 7.4)	H-2b, H-3a, H-3b
2b		1.94 (ddd, 13.4, 7.0, 1.0)	H-2a, H-3a, H-3b
3a	25.3 t	1.87 (br dd, 13.0, 7.4)	H-3b, H-2a, H-2b
3b		2.20 (ddd, 13.0, 13.1, 7.0)	H-3a, H-2a, H-2b
4	59.5 s		
5	62.6 d	3.22 (br s)	H-6
6	44.4 d	1.53 (br d, 11.6)	H-5, H-7
7	37.9 d	1.90 (m)	H-6, H-8a, H-8b, H-11
8a	24.3 t	1.08 (m)	H-7, H-8b, H-9a, H-9b
8b		1.72 (m)	H-7, H-8a, H-9a, H-9b
9a	30.1 t	1.47 (m)	H-8a, H-8b, H-9b, H-10
9b		1.58 (m)	H-8a, H-8b, H-9a, H-10
10	40.4 d	1.21 (m)	H-9a, H-9b, H-14
11	26.4 d	2.19 (m)	H-7, H-12, H-13
12	15.1 q	0.84 (d, 6.9)	H-11
13	21.3 q	1.00 (d, 6.9)	H-11
14	14.8 q	0.88 (d, 6.7)	H-10
15	24.6 q	1.38 (s)	
OH		3.43 (br s)	
